# Review of the 2017 WHO Guideline: Preventive chemotherapy to control soil-transmitted helminth infections in at-risk population groups. An opportunity lost in translation

**DOI:** 10.1371/journal.pntd.0006296

**Published:** 2018-04-26

**Authors:** Lorenzo Savioli, Marco Albonico, Denis Daumerie, Nathan C. Lo, J. Russell Stothard, Samuel Asaolu, Louis Albert Tchuem Tchuenté, Roy M. Anderson

**Affiliations:** 1 Department of Control of Neglected Tropical Diseases, World Health Organization, Geneva, Switzerland; 2 University of Torino, Torino, Italy; 3 Division of Epidemiology, Stanford University, Stanford, California, United States of America; 4 Department of Parasitology, Liverpool School of Tropical Medicine, Liverpool, United Kingdom; 5 Department of Zoology, Obafemi Awolowo University, Ife, Nigeria; 6 Faculty of Sciences, University of Yaoundé I, Yaoundé, Cameroon; 7 London Centre for Neglected Tropical Disease Research (LCNTDR), Department of Infectious Disease Epidemiology, Faculty of Medicine, Imperial College London, London, United Kingdom; University of Kelaniya, SRI LANKA

## Introduction

On 29 September 2017, the World Health Organization (WHO) published a new Guideline on the implementation of preventive chemotherapy (PC) programmes for the control of soil-transmitted helminthiasis (STH) infections [1]. The document supersedes two WHO publications that, in addition to STH, simultaneously addressed the control of schistosomiasis, onchocerciasis, and lymphatic filariasis (LF) by the coordinated implementation of regular, systematic, large-scale interventions that provide anthelminthic drug treatment to all individuals at risk of morbidity caused by these infections. Mass drug administration (MDA) is the key component in its implementation [2, 3]. With recent advances in understanding of the epidemiology of STH [4] and the changing funding landscape, the neglected tropical diseases (NTDs) community has eagerly awaited new guidance from WHO on PC. This is to set and safeguard the public health stage beyond the original targets and goals defined by the WHO NTD roadmap of 2012 that inspired the London Declaration and the commitment of several partners towards the control and/or elimination of NTDs [5]. The scientific rationale for this was summarised in an article published in the *Lancet* in 2012 [6] and also sustained by the series of the Disease Control Priorities by the World Bank [7]. Linked to this new knowledge, revised PC guidelines that incorporate novel information and address STH transmission and morbidity control were advocated in a number of publications [7, 8, 9]. The scientific backbone for the suggested changes was rigorous analyses that employed epidemiological data and models of transmission to derive optimum solutions for both morbidity and transmission control based on MDA coverage of various age groupings in affected communities [6].

WHO describes PC as the large-scale preventive treatment against helminthiasis and trachoma with safe, often single-dose, quality-assured medicines facilitated by several large-scale donations [10]. Without doubt, PC programmes represent a major public health intervention, delivering over 1 billion treatments every year (1.5 billion in 2016), and provide an essential standard of care for those at risk of infection or associated disease. PC policy is endorsed by a series of World Health Assembly (WHA) resolutions—including those of 2001, 2012, and 2013—and a series of informal consultations and Expert Committee reports [10, 11, 12, 13]. The development of the PC strategy by WHO has stimulated a large body of literature, reviewed by Gabrielli et al. as ‘the core intervention recommended by WHO for reducing morbidity and transmission of the four main helminthic infections, namely LF, onchocerciasis, schistosomiasis and soil-transmitted helminthiasis’ [14].

Regrettably, the opportunity to build on the previous momentum has been missed. We have identified five critical areas where the 2017 WHO guideline does not provide the necessary leadership:

1. Absence of guidance on the most urgent current issues, including school-based versus community-wide PC, drug combinations to increase anthelminthic efficacy, the use of new diagnostics, clearer protocols for monitoring anthelminthic efficacy/resistance, new prevalence thresholds for PC, morbidity control versus elimination end-game targets.
2. No mention of the need to accurately monitor drug coverage and individual compliance at successive rounds of MDA.
3. Insufficient discussion about available data on deworming and what this tells us about successes and failures, and approaches to address any data deficits.
4. Absence of any WHO recommendation for schistosomiasis, onchocerciasis, and LF, as the new guideline only considers STH and ignores opportunities for integration.
5. Inadequate pragmatic guidance for programme managers on the implementation, monitoring, and evaluation of PC STH programmes, or the integration of PC interventions needed to revise the threshold for PC when moving from control to elimination, looking beyond the 2020 deadline to reach the Sustainable Development Goals (SDGs) for NTDs by 2030.

## The new 2017 guideline: Preventive chemotherapy to control STH infections in at-risk population groups

In developing this new guideline, WHO has involved the NTD department, the WHO Evidence and Programme Guidance unit in charge of the clearance of WHO guidelines, and the Nutrition department. It has also engaged those involved in the Cochrane review on deworming, which has concluded there is little evidence to support PC for control of morbidity caused by helminthic infections, generating debate in the PC community [15]. The WHO Evidence and Programme Guidance unit operates following the Cochrane Collaboration procedures, as mentioned on page 2 of the new guideline [16]. This approach is a marked shift from the traditional methodology based on WHO Expert Committees informed by a thorough review of peer-reviewed evidence from randomised control trials and observational studies, expert advice, and on-the-ground experience. This more recently established practice creates the risk of relying solely on a specific approach that is not universally accepted in decision making for grant allocation or selection of public health interventions [17]. In the last 2 decades, a large group of scientists, public health experts, managers of control programmes, and indeed WHO staff members have argued a fundamental methodological flaw has contributed to the Cochrane systematic review and meta-analysis on deworming because of their protocol that only allows data from randomized control trials and does not incorporate other sources of evidence and experience [15, 18]. In particular, for helminth infections, the Cochrane reviews for STH have not explicitly taken into account the known link between the intensity of infection and morbidity with reliance in the reviews on prevalence instead of intensity. Helminth parasite distributions are highly aggregated, where most worms are harboured by few people; as such, the morbidity impact in a few can be hidden by sampling the whole population. Further articles have provided broader critique of many reasons why randomised trials of short duration, often in low-burden settings with limited sample size, can be problematic for measurement of many key health outcomes [7, 8, 9].

The new PC guideline includes information on the evidence and strength of each of the recommendations. These parameters were agreed upon by secret ballot, with abstentions prohibited. On page 43, a primary and secondary decision rule is described with a report on the approach for approving the document, with the voting forms to be kept on file in WHO for up to 5 years.

This long process to grade evidence according to the *Cochrane Handbook* has generated three recommendations, described in less than one page, that can be summarized as

1. A strong recommendation with low-quality evidence for regular treatment with single-dose albendazole or mebendazole for young, preschool, and school-age children living in areas where prevalence of STH is more than 20%. Young children have never been previously identified by WHO and could represent an age overlap, complicating reporting by control programmes.
2. A strong recommendation with moderate-quality evidence for treatment with single-dose albendazole or mebendazole for girls and nonpregnant women living in areas where prevalence of STH is more than 20%.
3. A conditional recommendation with moderate-quality evidence for benzimidazole treatment of pregnant women living in areas where hookworm and *Trichuris trichiura* prevalence is more than 20% and where anaemia is a severe public health problem.

Of the 29 outcomes listed in the annex of the new guideline (e.g., haemoglobin, cognition, weight gain, height gain, etc.), 51% are low of very low-quality evidence, 37% moderate, and only 10% (3 out of 29) high. A footnote on the ‘quality of evidence’ on page 29 states that ‘According to GRADE, moderate-quality evidence indicates we are moderately confident in the estimate of the effect and the true effect is likely to be close to the estimate of the effect, but there is a possibility that it is substantially different’. This dichotomous ‘true or false’ approach is inappropriate given that there seems to be no relationship between quality of evidence and strength of the recommendation and important heterogeneities that complicate a binary conclusion (e.g., different epidemiology, drug efficacy, and time horizons of trials).

## Conclusions and lessons learned

In summary, the new guideline has led to the reassessment—a majority with low or very low evidence—of the validity of the three longstanding WHO recommendations regarding large-scale PC treatment for children, girls, and women, adding also the new group of young children. The methodology used has been previously critiqued by the WHO NTD department and the academic and NTD programmatic community [15]. This has resulted in a guideline that lacks pragmatic advice for programme managers and public health experts on the implementation, monitoring, and evaluation of PC STH programmes and the integration of PC interventions.

The disconnect between the strength of the recommendation and the document’s blanket judgment on the quality of evidence does not help NTD programme managers balance their meagre budgets and resources, and risks causing confusion. Only time will tell how the new and complex guideline, which now supersedes the earlier ones dealing with all helminthiasis and not just STH, will be perceived by the Ministries of Health in endemic countries currently pressed to reach the WHO NTD PC roadmap targets by 2020.

The independent selection of WHO panels of experts who can assess a broad range of evidence—including randomised trials, observational studies, and expert opinion, when appropriate—will allow for a more holistic evaluation of evidence for optimal and collaborative policy decision making. This is critical, especially for the control of NTDs, in which implementing randomised control trials for many meaningful health outcomes is not feasible. The damaging effects of this PC guideline are starting to have a negative impact. On 4 October 2017, a *BMJ* article commented that ‘WHO continues to advise deworming of millions of children, despite “lacking benefits”‘ [19]. This article does not recognise that the problem is not in the PC strategy, but in the analytical methods and data employed to look at associations between helminth burden and morbidity.

The Cochrane methodology is but one approach for evaluating the public health relevance of NTD control recommendations. Its weakness for helminth infections lies in what epidemiological measure of infection is employed. Intensity and its link to morbidity, plus their aggregated distributions—or uneven worms per person—in an infected community is the most rigorous approach but is not employed in the published reviews. Other observational longitudinal studies come to different conclusions. Reconciling these differences is an essential prerequisite before any change is made to the current large-scale treatment programmes.

In 2004, the *Lancet* encouraged the international public health community to embrace ‘Thinking beyond deworming’ as an essential element to improve the health of the world’s poorest people [20]. Rather than moving forward to explore new strategies (e.g., community deworming, drug combinations) or complementary interventions (e.g., water, sanitation, and hygiene [WASH]), these guidelines have missed an opportunity to move forward [21]. The new 2017 guideline is, in our view, a disappointing return to the past pre-2004 instead of looking at the future beyond 2020.

We suggest that WHO urgently sets up a working group to produce a second edition of the 2006 PC in human helminthiasis manual for health professionals and programme managers. Until such time, we advise the NTD community to continue to implement the 2006 manual and 2011 guide that are supported by WHA resolutions and judge this new guideline in this light.

What is needed today are recommendations on the role of PC beyond morbidity control and the approaching 2020 targets of the NTD roadmap. Some critical contemporary topics based on new evidence that may improve the five critical areas mentioned in the ‘Introduction’ could be

1. Establishing or redefining clear endemicity thresholds for all possible types of PC diseases (notably onchocerciasis, LF, loiasis, schistosomiasis, STH, and trachoma) in exposed populations and defining proper PC combinations to be delivered (addresses critical areas 1 and 4).
2. Consensus on treatment of target age groups such as children, girls, and women of childbearing age and adults for all PC diseases (addresses critical areas 1 and 4).
3. Recommendations on new field-applicable diagnostics for screening and treating target groups and for monitoring the impact of PC intervention on morbidity and transmission (addresses critical area 1).
4. Guidance on the use of drug combinations to increase anthelminthic performance and mitigate risks of development of drug resistance for both first-line and second-line treatments (addresses critical area 1).
5. Clear protocols for monitoring anthelminthic efficacy in front-line treatments (addresses critical area 1).
6. Critical evaluation of coverage data and drug needs, including the delivery capacity of endemic countries, to identify areas to improve health system and drug access for STH and other NTDs (addresses critical areas 2 and 3).
7. Establishing new prevalence thresholds to decide when to start and when to scale down and halt PC for guiding STH and schistosomiasis elimination targets, assuming this has already been undertaken for onchocerciasis and LF (addresses critical area 5).

In the last decade, billions of US dollars have been invested by Member States and the pharmaceutical industry to implement PC programmes under the leadership of WHO. Billions of the world’s poorest have benefitted from PC. The international community needs the strong leadership of WHO to generate pragmatic guidance towards reaching the NTDs UN SDGs by 2030 [22].
